# Association between Gut Dysbiosis and the Occurrence of SIBO, LIBO, SIFO and IMO

**DOI:** 10.3390/microorganisms11030573

**Published:** 2023-02-24

**Authors:** Michalina Banaszak, Ilona Górna, Dagmara Woźniak, Juliusz Przysławski, Sławomira Drzymała-Czyż

**Affiliations:** 1 Department of Bromatology, Poznan University of Medical Sciences, Rokietnicka 3, 60-806 Poznan, Poland; 2 Poznan University of Medical Sciences Doctoral School, Poznan University of Medical Sciences, Bukowska 70, 60-812 Poznan, Poland

**Keywords:** gut microbiota, dysbiosis, bacteria, bacterial overgrowth, abdominal pain

## Abstract

Gut microbiota is the aggregate of all microorganisms in the human digestive system. There are 10^14^ CFU/mL of such microorganisms in the human body, including bacteria, viruses, fungi, archaea and protozoa. The Firmicutes and Bacteroidetes bacteria phyla comprise 90% of the human gut microbiota. The microbiota support the healthy functioning of the human body by helping with digestion (mainly via short-chain fatty acids and amino acids) and producing short-chain fatty acids. In addition, it exhibits many physiological functions, such as forming the intestinal epithelium, intestinal integrity maintenance, the production of vitamins, and protection against pathogens. An altered composition or the number of microorganisms, known as dysbiosis, disrupts the body’s homeostasis and can lead to the development of inflammatory bowel disease, irritable bowel syndrome, and metabolic diseases such as diabetes, obesity and allergies. Several types of disruptions to the gut microbiota have been identified: SIBO (Small Intestinal Bacterial Overgrowth), LIBO (Large Intestinal Bacterial Overgrowth), SIFO (Small Intestinal Fungal Overgrowth), and IMO (Intestinal Methanogen Overgrowth). General gastrointestinal problems such as abdominal pain, bloating, gas, diarrhoea and constipation are the main symptoms of dysbiosis. They lead to malabsorption, nutrient deficiencies, anaemia and hypoproteinaemia. Increased lipopolysaccharide (LPS) permeability, stimulating the inflammatory response and resulting in chronic inflammation, has been identified as the leading cause of microbial overgrowth in the gut. The subject literature is extensive but of limited quality. Despite the recent interest in the gut microbiome and its disorders, more clinical research is needed to determine the pathophysiology, effective treatments, and prevention of small and large intestinal microbiota overgrowth. This review was designed to provide an overview of the available literature on intestinal microbial dysbiosis (SIBO, LIBO, SIFO and IMO) and to determine whether it represents a real threat to human health.

## 1. Introduction

**Figure 1:**
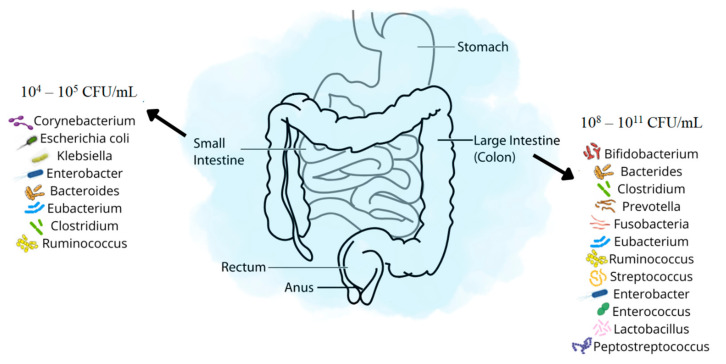
Distribution of particular types of bacteria in the digestive system (based on [3,4,6]).

Gut microbiota is the aggregate of all microorganisms in the human digestive system. They include bacteria, viruses, fungi, archaea and protozoa. The population of organisms varies according to the section of the digestive system. In the stomach and the duodenum, because of the acidity of gastric juices and short passage time, their numbers are lowest, between 10^3^ and 10^4^ CFU/mL (CFU/mL—colony forming units per millilitre). The stomach is an unfavorable environment for the growth of bacteria. Nevertheless, microbiological and molecular tests have revealed that the bacteria *Firmicutes* (*Lactobacillus*, *Streptococcus*, *Clostridium*, *Veilonella*), *Proteobacteria* (*Escherichia*), *Actinobacteria* (*Bifidobacterium*), and also *Candida fungi* are found in the stomach [1,2]. Simultaneously, the most common pathogen in the stomach is *Helicobacter pylori*. Some studies show that it is present in over 50% of the population worldwide. However, the most significant number of bacteria is found in the distal part of the small and large intestines (ileum—10^8^ CFU/mL, colon—10^11^ CFU/mL) (Figure 1) [3,4]. The number of microorganisms in the human body is estimated at 10^14^ CFU/mL. The latest research suggests roughly the same number of bacterial and human cells [5].

As mentioned above, the microbiome primarily consists of bacteria, viruses and fungi.

The 160 or so species of microorganisms include *Firmicutes*, *Actinobacteria*, *Pseudomonadota*, *Fusobacteria* and *Verrucomicrobia*. *Firmicutes* and *Bacteroidetes bacteria* phyla dominate and make up 90% of the human gut microbiota. Over 200 different genera of bacteria represent the *Firmicutes phylum*. These include *Lactobacillus*, *Clostridium*, *Enterococcus* and *Ruminococcus*. The *Bacteroidetes phylum* is mainly characterized by the *Bacteroides* and *Prevotella* genera [7].

Viruses are also part of the intestinal microbiota. They may have immune functions. We distinguish viruses that infect host cells and bacteriophages that affect bacteria, the latter being the most popular among intestinal viruses. At least 10^9^ virus-like particles have been isolated from one gram of faeces. Many of them still need to be identified. The most common viruses in the gut microbiota are double-stranded DNA viruses of the order *Caudovirales* (*Podoviridae*, *Siphoviridae*, and *Myoviridae*) and single-stranded DNA bacteriophages (*Microviridae*) [2,8].

Fungi found in the human intestine have yet to be precisely established, despite the growing interest in the intestinal mycobiota. The most common types of fungi are *Candida*, *Cladosporium*, *Cryptococcus* and *Saccharomyces*, but in many stools samples can be also found *Malassezia* spp., *Eurotiales* spp., *Botrysphaeriales* spp. and *Filobasidiales* spp. In the large intestine, 10^2^–10^4^ fungi cells per one gram of faeces samples also have been observed [2,9]. Hoffman et al. [10] determined that *Candida* and *Saccharomyces* fungi positively correlate with *Methanobrevibacter archaea* and *Prevotella bacteria*. These microorganisms were most often found in people on a high-carbohydrate diet. On the other hand, people with a lot of fatty acids in their diet show an increased number of *Candida*. Researchers concluded that *Prevotella*, *Ruminococcus*, *Candida* and *Methanobrevibacter* exhibit syntrophy. *Prevotella* and *Rumnicoccus* ferment the sugars *Candida* produces, while *Methanobrevibacter* uses bacterial fermentation products to synthesize methane and carbon dioxide.

Most bacteria, viruses, and fungi in gut microbiota composition still need to be identified, and further research is fundamental. The Human Microbiome Project (HMP) was the breakthrough in intestinal microbiota knowledge. Based on 16S rRNA sequencing, the gut microbiota diversity has been described. The human microbiome was assessed at the level of the nucleotide sequence of the genomic DNA of microorganisms (metagome), the nucleotide sequence of mtRNA (mitochondrial RNA) (metatranscriptome), the synthesis of bacterial proteins (metaproteome), and the products of microbial metabolism (metabolome) originating from different places in the human body: the skin, oral cavity, digestive tract, and genitourinary system. Differences in the microbiota depend on the human population and its various properties, genotype, age, diet, living environment, and the health status examined. Despite advanced research, human gut microbiota composition has yet to be fully understood. Future metagenomic studies may help identify the microbiota’s composition and, thus, dysbiosis [2,11,12].

As previously mentioned, the human digestive tract (especially the small and large intestines), due to its functions, the availability of nutrients and the specificity of its structure, is an unusual place for the development and growth of microorganisms. However, the first part of the small intestine—the duodenum—is not a favorable environment for developing microbiota. The number of bacteria in the small intestine gradually increases. In the jejunum is 10^5^ CFU/mL, of which *Bacteroides*, *Lactobacillus* and *Streptococcus* are mainly present. In the ileum, the number of organisms increases to 10^8^ CFU/mL, mainly consisting of *Bacteroides*, *Clostridium*, *Enterococcus*, *Lactobacillus*, *Veillonella*, and *Enterobacteriaceae*. The large intestine is the richest reservoir of intestinal microorganisms—the mass of bacteria in the intestine is 1.5–2 kg. In the large intestine, the majority of bacteria is strictly anaerobic (*Bacteroides*, *Clostridium*, *Ruminococcus*, *Fusobacterium*, *Butyrivibrio*, *Peptostreptococcus*, *Eubacterium* and *Bifidobacterium*), aerobic and relatively aerobic (Gram-negative from the *Enterobacteriaceae* family, Gram-positive from the *Lactobacillus*, *Enterococcus* and *Streptococcus* family) and includes small amounts of fungi, viruses and archaea [13].

The relationship between humans and their microbiota enables the proper functioning of the human body. It exhibits several physiological functions, which we divide into trophic, metabolic and immunological. The trophic is responsible for nutrition (butyric acid), the forming of epithelial cells, and stimulating the synthesis of mucins accountable for protecting the epithelium against pathogens. The metabolic functions are aiding digestion (mainly amino acids), producing B and K vitamins and short-chain fatty acids, assimilating vitamins and nutrients, and reducing fat absorption. Microbiota is an essential element of the body’s immune system: it secretes substances that inhibit the development of unfavorable bacteria and viruses (bacteriocins, hydrogen peroxide, lactoperoxidases, organic acids that lower pH) [6,14,15,16]. Due to the human microbiota’s many functions, any rapid change in its composition may be associated with various disease symptoms. However, unambiguously linking individual diseases with changes in the intestinal microbiome is not easy because these changes can be both the cause and the effect of disease symptoms. Hence, the proper colonization of the digestive tract seems to be extremely important.

According to the ‘sterile uterus’ hypothesis, microbial colonisation of the foetal gut does not begin until birth [17]. It should be mentioned, however, that some studies show that the colonisation of the human gastrointestinal tract starts during foetal life through contact with the maternal metabolome and not directly with the mother’s microflora. Despite some ambiguities regarding the timing of the onset of gastrointestinal colonisation, it is still recognised that the main factors determining the composition of the acquired microbiota are the duration of pregnancy, delivery method, diet, environment, medications taken, and hospitalisation [18,19]. The microbiota is significantly less diverse in premature babies (born before 37 weeks gestation) than in full-term babies. The increased colonisation of pathogenic *Proteobacteria* and reduced numbers of *Bifidobacterium*, *Bacteroides* and *Atopbium* anaerobes were also noted in young patients. Immaturity of the digestive system or other environmental factors, such as prolonged hospitalisation or medication, may cause this [7,20,21]. Colonisation is also affected by the type of delivery. Newborns born by natural birth have a microbial composition in the digestive tract similar to that of the mother’s vaginal microbiota. *Lactobacillus*, *Prevotella* and *Sneathia* genera bacteria are dominant. Newborns born by caesarean section have a less diverse gut microbiome and acquire bacteria mainly from the hospital environment and the mother’s skin. These include *Staphylococcus*, *Corynebacterium* and *Propionibacterium* spp. genera [22,23,24]. Research shows that individuals born by caesarean section in the future may be exposed to an increased risk of asthma, connective tissue disease, juvenile arthritis, inflammatory bowel disease and childhood obesity [25,26]. The diet also influences the microbiota composition, so breastfed infants have a more diverse and prosperous intestinal microbiota, particularly in *Bifidobacterium* spp., and exhibit a lower abundance of *Clostridium* difficile and *Escherichia coli* compared to children fed breast milk replacement products [27]. At around three years of age, the gut microbiota of children becomes similar to that of adults in terms of composition and diversity. The microbiota of older people (over 70 years of age) may show less variety, with a notable decrease in *Bifidobacterium* spp. and an increase in *Clostridium* and *Proteobacteria* [28]. Research shows that reducing the levels of Bifidobacteria, which are responsible for stimulating the immune system, can result in general mild inflammation and malnutrition in older people [29].

Dysbiosis encompasses any proliferation, change in the composition or disappearance of the microbiota. This condition disrupts the body’s homeostasis and can lead to the development of inflammatory bowel disease, irritable bowel syndrome, and metabolic diseases such as diabetes, obesity and allergies. Research shows that overweight and obese people have fewer gut microbial genes, translating into a lower total bacterial population than individuals with the correct body weight. In addition, the aggregate gut microbiota negatively correlates with metabolic parameters such as body mass index (BMI), body fat percentage, insulin, leptin, C-reactive protein concentrations, and the homeostasis model assessment of insulin resistance (HOMA-IR). Impaired immunity due to reduced beneficial microorganisms, impaired gut barrier function, and the influx of pathogens can lead to autoimmune diseases [30,31,32,33]. Environmental factors, including geographical location, diet, smoking, alcohol and medications, are just some factors that contribute to changes in the composition and quantity of the gut microbiota. Antibiotics disrupt the microbiota’s short- and long-term balance, resulting in an imbalance in population sizes and diversity [14,34]. Changes in the gut microbiota due to the type of antibiotics taken are shown in Table 1.

Recently, there has been growing interest in the impact of the gut microflora and its dysbiosis on the human body. It has led to several types of disruptions to the gut microbiota balance being identified: SIBO (*Small Intestinal Bacterial* Overgrowth), LIBO (*Large Intestinal Bacterial* Overgrowth), SIFO (*Small Intestinal Fungal* Overgrowth, and IMO (*Intestinal Methanogen* Overgrowth).

## 2. Small Intestinal Bacterial Overgrowth—SIBO

Small intestinal bacterial overgrowth is a disorder that has been extensively studied in recent years. SIBO can be defined as the presence of colon-specific bacteria in the small intestine equal to or greater than 10^5^ CFU/mL. It is sometimes called a change in the small intestine’s standard balance of individual gut microbiota species. Patients with SIBO produce hydrogen due to the fermentation of consumed carbohydrates. Bacteria characteristic of small intestinal bacterial overgrowth include *Streptococcus*, *Staphylococcus*, *Bacteroides*, and *Lactobacillus*. Among *Enterobacteriaceae* family pathogens, an increase in the number of bacteria of the *Escherichia*, *Klebsiella* and *Proteus* genera is mainly noted. General gastrointestinal problems such as abdominal pain, bloating, gas, diarrhoea and irregular bowel movements are the main symptoms of SIBO. These problems can lead to malabsorption, resulting in nutritional deficiencies, anaemia or hypoproteinaemia. Increased lipopolysaccharide (LPS) permeability, stimulating the inflammatory response and resulting in chronic inflammation, has been identified as the leading cause of SIBO. In addition, irregularities in the structure and function of the intestinal wall, excessive concentrations of ghrelin, leptin or TMAO (trimethylamine N-oxide), the presence of pro-inflammatory cytokines and increased gastric pH may also cause SIBO. Low ileocecal valve pressure is often associated with the development of SIBO and is a predisposing factor. The gold standard for diagnosing SIBO uses a jejunum aspirate and culture. However, due to the invasiveness of the test, breath tests (BTs) with glucose (GBT) or lactulose (LBT) are more commonly used. A concentration of ≥10^3^ CFU/mL in a culture or an increase of ≥20 ppm in the concentration of hydrogen compared to baseline is synonymous with a SIBO diagnosis [36,37,38,39].

SIBO often accompanies other digestive system diseases as well as other conditions. Numerous studies describe the simultaneous occurrence of SIBO and IBS (irritable bowel syndrome) [40,41,42,43]. Both disorders stimulate the immune system, which increases pro-inflammatory cytokines in the intestinal mucosa and may increase its permeability [36]. Research also indicates that bacterial overgrowth is more common for people suffering from IBS-C (Irritable Bowel Syndrome with Constipation) than for those suffering from IBS-D (Irritable Bowel Syndrome with Diarrhoea) [44]. A higher incidence of SIBO is also associated with Inflammatory bowel diseases and Crohn’s disease in particular [45,46,47,48]. Various fistulas, stenoses and surgical procedures significantly impact intestinal motility. They can also lead to microbiota dysbiosis [49]. Research suggests that SIBO also accompanies coeliac disease. Intestinal motility problems during celiac disease may have been the cause of bacterial overgrowth [50,51].

Metabolic diseases are complex disorders associated with the occurrence of chronic, long-term, low-intensity inflammation, which impacts the microbiota. Abnormal leptin and ghrelin levels have a negative impact on intestinal peristalsis. A decrease in *Bacteroides bacteria* relative to the *Firmicutes bacteria* observed in obese people compared to slim people may lead to an excessive calorie intake, increased production of short-chain fatty acids, and adipocyte hyperplasia, which consequently increases LPS and endotoxin intestinal permeability, which causes the inflammation. The co-occurrence of obesity and SIBO thus constitutes a vicious circle. Interestingly, research highlights that intestinal dysbiosis is not only more common among people with obesity but also among those with metabolic syndrome [52,53]. Diabetes patients often develop gastrointestinal disorders [54]. The prevalence of SIBO amongst type 1 and type 2 diabetic patients is significantly higher than in the general population. Intestinal dysbiosis can cause diabetes and vice versa. Dysbiosis may damage insulin receptors by stimulating the immune system and causing increased cytokine production. On the other hand, autonomic neuropathy and hyperglycaemia, as complications of diabetes, can impair gastrointestinal peristalsis, creating favourable conditions for bacterial overgrowth [55,56,57,58].

In addition to the diseases mentioned above, the co-occurrence of SIBO has been identified in:

- non-alcoholic fatty liver disease [59,60,61],
- cirrhosis [62],
- chronic pancreatitis [63],
- obesity [64],
- cystic fibrosis [65,66],
- heart failure [67,68],
- hypothyroidism [69],
- Parkinson’s disease [70,71,72],
- depression [73,74,75],
- systemic sclerosis [76,77]
- chronic renal failure [78,79].

A wide range of research papers confirms the multifactorial impact of SIBO on gastrointestinal, cardiovascular, endocrine, neurological, nephrological and bone diseases. Despite this, more research is still needed to identify effective treatments and prevent small intestinal bacterial overgrowth [36].

As mentioned, a disease entity particularly predisposing to SIBO is cystic fibrosis. Cystic fibrosis (CF) is one of the most frequently occurring genetically conditioned diseases inherited in an autosomal recessive manner [80]. The cause of the disease is the mutations of the CFTR gene (Cystic Fibrosis Transmembrane Regulator) located on chromosome no. 7 [81]. The product of this gene is the CFTR protein, present on the apical surface of the epithelial cells of the respiratory system, pancreas, intestine and sweat glands, which acts as a chloride channel. A defective structure/function or a lack of the chloride channel contributes to the production and deposit of thick, sticky mucus in the organs mentioned above. It should be emphasised that CF is a systemic disease, manifesting primarily in chronic broncho-pulmonary disease and pancreatic exocrine secretion insufficiency (PESI), with subsequent disorders of digestion and absorption [82,83]. The main symptom of PESI is fat diarrhoea. Its intensity depends on the amount of fat consumed, residual pancreatic capacity, comorbid gastrointestinal motility disorders, atrophy of the small intestine mucosa, changes in intestinal pH, and intestinal bacterial overgrowth syndrome [84,85]. Moreover, the presence of large amounts of dense, poorly hydrated mucus in the intestinal lumen in case of patients with CF additionally promotes bacterial overgrowth and, as a result, the onset of SIBO. At the same time, the need for long-term, chronic intake of antibiotics and proton pump inhibitors (PPI) predisposes patients with CF to dysbiosis. It is worth noting that CF is associated with the coexistence of numerous additional diseases and gastroenterological-hepatological complications [86,87,88].

It is worthy of note that the course of SIBO in patients with cystic fibrosis may differ slightly from other patients. The bacterial flora of the large intestine in cystic fibrosis patients is unable to produce hydrogen after lactulose administration more often than in healthy people [89]. Therefore, in the study by Lisowska et al. [90], it was assumed that a similar situation might occur with the small intestine. SIBO was found in over 37% of patients with CF. In almost every third patient, the measurement of methane was the basis for making the diagnosis. Therefore, in CF patients, it is advisable to replace the currently commonly used hydrogen breath test with a hydrogen-methane test in the diagnosis of SIBO. Otherwise, there is a high risk of false-negative results. As a continuation of the above study, it was also shown that as a result of antibiotic therapy, a significant reduction in hydrogen and methane excretion was obtained, reflecting a decrease in bacterial colonisation of the small intestine. A significantly more substantial effect was obtained for oral antibiotic therapy (ciprofloxacin (35–50 mg/kg/day)) compared with intravenous therapy (ceftazidime and amikacin in therapeutic doses (150–250 mg and 20–35 mg /mg/kg/day, respectively)) [91].

Several treatments for SIBO have been described in the literature. Antibiotics are widely prescribed, although evidence supporting their use is of low to moderate quality. SIBO often recurs after antibiotic therapy [39]. In a study by Lauritano et al. [92] on 80 patients, recurrence of SIBO symptoms was observed in 12.6% of the subjects after three months, 27.5% after six months, and 43.7% after nine months. Recommended antibiotics, doses, and their efficacy are shown in Table 2.

Non-pharmacological methods also help in the treatment of small intestinal bacterial overgrowth. Research suggests reducing fermenting foods and avoiding fibre-rich products, polyols, sweeteners, and prebiotics. This diet-based approach is called the FODMAP (Fermentable Oligosaccharides, Disaccharides, Monosaccharides And Polyols) diet. McIntosha et al. [93] conducted a 3-week study on IBS patients randomly assigned to a low (n = 20) or high (n = 20) FODMAP group. The low FODMAP group exhibited a reduction in the severity of IBS symptoms (*p* < 0.001), an increase in the number and diversity of *Actinobacteria,* and a slight decrease in hydrogen production. Such changes were not observed in the group on a FODMAP-rich diet. According to the available literature and the American College of Gastroenterology (ACG), the use of probiotics needs to be justified in the treatment of SIBO on account of low-quality research on too small a population. The situation with faecal microbiota transplantation (FMT) is similar. Insufficient evidence supports the efficacy of treating SIBO using this method [39].

## 3. Large Intestinal Bacterial Overgrowth—LIBO

The large intestine is the largest reservoir of gut microbiota. The term LIBO (large intestinal bacterial overgrowth) has emerged in scientific circles and at conferences. Such a statement has yet to be figured out in any medical article. It may be a catchy title used to attract attention.

There is no doubt that the gut microbiota of the large intestine can suffer from dysbiosis. However, only exceptional cases in which this has occurred have been described. Drago et al. [94] assessed the composition of the colonic intestinal microbiota of people subjected to colonoscopy. These were screening tests for colorectal cancer, and the results for all subjects were average. Stool samples were collected from the subjects one month before and one month after the colonoscopy. Patients were asked to drink a standard polyethene glycol-based solution (4 litres) to prepare for the study. After ingesting such a large volume of fluids and following an endoscopic examination, the stool samples examined showed a reduction in *Clostridia* and *Firmicutes bacteria* and an increase in *Proteobacteria* compared to the pre-examination sample. A decrease in *Lactobacillus* and a significant rise in *Enterobacteriaceae* were also observed. In addition, there was a fourfold increase in *Streptococcaceae* 30 days after testing, which may indicate an increase in intestinal permeability and the presence of proteases in the stool. Stool samples collected from patients 30 days after the examination showed an almost complete return of the microbiota composition to its pre-colonoscopy state. The short-term significant intestinal dysbiosis described by the researchers was similar to that observed during severe diarrhoea in children in developing countries [95,96].

Studies also indicate that patients who experienced short-term dysbiosis, e.g., due to cleansing the large intestine before colonoscopy, developed abdominal pain and discomfort for up to 30 days after the endoscopy [96]. It may suggest that gastrointestinal disorders accompanying LIBO could be similar to those for SIBO (abdominal pain, bloating, gas and abnormal bowel movements).

However, can diet itself, and not just diseases, knock the gut microbiota out of balance? Selmin et al. [97] conducted research on mice, giving researchers ample room to manoeuvre when applying a similar idea to human research. The study analysed the effects of a typical western diet (WD) (58.4%, 27.8% and 13.7% of energy from carbohydrates, fats and proteins, respectively) and an isocaloric and isoprotein soy oil-rich diet (n-6HFD—a high-fat diet rich in omega six fatty acids) (50% and 35.9% of energy from aggregate fat and carbohydrates) on the gut microbiota. The researchers noted changes in animal body weight—animals on the high-fat diet weighed 25% more than those on the WD diet. Histopathological imaging of the colon showed that the n-6HFD diet increased the incidence of inflammation and irregularities in the intestinal tissue (hyperblastic lesions and mild fibrosis). This was also confirmed by a cyclooxygenase-2 (COX-2) expression test induced by the n-6HFD diet. It is worth noting that COX-2 overexpression is a marker of inflammatory diseases [98]. The gut microbiota was also disrupted. The n-6HFD group showed a decrease in *Firmicutes*, *Clostridia* and *Lachnospiraceae,* and a parallel increase in *Bacteroidetes*, *Deferribacteres* and *Verrucomicrobia*. In addition, there was also an increase in the numbers of the pro-inflammatory *Mucispirillum schaedleri* and *Lactobacillus murinus* bacteria. That study provides essential information regarding the diet and changes in the gut microbiota, which may be relevant to potential non-pharmacological LIBO treatments [97].

Of course, it is not only a change in diet that can lead to dysbiosis. In line with what was mentioned earlier, antibiotic therapy is one of the most significant factors leading to a change in the composition of the microbiota. Very broad-spectrum antibiotics are often used to treat various types of infection, and many of these are orally administered antibiotics. As a result, the gastrointestinal microbiota is exposed to high concentrations of antibiotics [99]. Antibiotic therapy reduces the total number of bacteria in the gastrointestinal tract, significantly alters its composition, and drastically reduces the species diversity of the gastrointestinal microbiome [100,101]. *Firmicutes bacteria* have been observed to be particularly sensitive to antibiotics. According to Antonopoulos et al. [100], the proportion of this group of bacteria decreased from 74% to only 7.5% after administering antibiotics. At the same time, the balance of bacteria of the *Bacteroidetes phylum* decreased from 23.3% to 16.8%. At the same time, the authors noted a very sharp increase in the ratio of the *Proteobacteria phylum* (from 1.1% to 75.5%). Moreover, although the microbiome returns to a state similar to that before treatment after discontinuation of antibiotic therapy, Jernberg et al. observed significant differences in microbiota composition for as much as two years after antibiotic therapy [102,103]. This suggests that, despite the main composition of the microbiome being restored, returning to the state in which it was before antibiotic treatment may be difficult to achieve.

Dysbiosis can also apply to fungi, particularly in polyps and colorectal cancer patients. In their research, Gao et al. [104] demonstrated reduced fungal diversity in patients with polyps, increased Ascomycota to Basidiomycota, and relatively more Trichosporon and *Malassezia* opportunistic fungi. These imbalances may favour colorectal cancer progression. Can we, therefore, speak of an extensive intestinal fungal overgrowth (LIFO)? The subject of gut microbiota in human health and disease requires more research.

## 4. Small Intestinal Fungal Overgrowth—SIFO

Small intestinal fungal overgrowth is a dysbiosis involving excess fungi in the small intestine. This condition is accompanied by gastrointestinal disorders such as those observed for SIBO (abdominal pain, gas, bloating and diarrhoea). In the past, SIFO was diagnosed in HIV-positive patients, during steroid therapy and antibiotic therapy in cancer patients, and in those with poorly managed diabetes. It was due to a reduction in immunity (abnormalities in neutrophil chemotaxis, adhesion and intracellular pathogen-killing capacity) that facilitated fungal overgrowth [105]. It caused systemic fungal infections, including small intestinal fungal overgrowth. People with fungal overgrowth and comorbidities such as diabetes or those being treated with antibiotics or in chemotherapy may experience more exacerbated symptoms—watery diarrhoea 8–10 times a day, mucus in the stool, urgency, bloating, or severe abdominal pain. However, recent studies show that SIFO can affect healthy and immunocompetent individuals and cause unpleasant gastrointestinal complaints [106,107]. Interestingly, immunocompromised individuals with fungal overgrowth also reported chest pains, belching, indigestion and gas.

People with weakened immune systems, particularly those infected suffering from HIV, cancer, or diabetes, or those subject to cancer chemotherapy, on immunosuppressants, steroids or antibiotics, are more likely to develop SIFO. Organ transplant patients, children, the elderly and hospitalised patients may be at risk of fungal overgrowth. However, we do not know why SIFO affects healthy people with normal immunity. Studies show that using proton pump inhibitors may impair small intestine peristalsis and be a predisposing factor to developing SIFO [108].

A study on healthy individuals showed that approximately 70% had *Candida* albicans in the stomach and small intestine at low concentrations of up to 10^2^ CFU/mL. However, whether this is clinically relevant to patients with gastroenterological symptoms has yet to be established. It is also uncertain whether fungal-targeted treatments, without a known predisposing factor, are effective and guarantee remission [109]. A study by Jacobs et al. showed that out of 150 patients with gastrointestinal disorders of unknown origin examined endoscopically and radiologically with negative results, 24 were diagnosed with SIFO, 38 had SIBO and 32 had mixed SIBO and SIFO [108]. Similar studies have found that 25.3% (38/150) of patients with unexplained chronic gastrointestinal symptoms have SIFO. The authors also noted that the only method of identifying SIFO is by the culture of aspirated intestinal juice [110]. A diagnosis can be made if the saccharomycete count exceeds 10^3^. In the past, circulating IgG immune complexes containing *Candida* antibodies and IgG/IgA/IgM class *Candida* antibodies tests were used. Still, these parameters can only suggest SIFO and may be used to assess the efficacy of therapy. None of them, however, allow a specific diagnosis to be made. Fungi may also be present in the faeces, but stool culture testing for *Candida* cannot diagnose small intestinal fungal overgrowth in healthy individuals [109,110].

Treating SIFO is complex and needs to be tailored to each patient. Comorbidities, the severity of candidiasis, intolerance to antifungal treatment, and the patient’s immunity and clinical stability must be considered. Some strains of *Candida* can be resistant to azole antifungal agents. Establishing what type of strain is present in the patient is also worthwhile. Fluconazole and nystatin may be effective in treating small intestinal fungal overgrowth. However, the dosage and the appropriate treatment duration for people with SIFO have yet to be established, which may result in complications and a lack of improvement in the patient’s condition. Further research is needed into the pathophysiology, diagnosis, and treatment of SIFO [106,111].

## 5. Intestinal Methanogen Overgrowth—IMO

Intestinal methanogen overgrowth is a relatively new issue. The last name for methanogen overgrowth, or ‘methane-SIBO’, is incorrect, as bacteria are responsible for SIBO and prokaryotic organisms—archaeons, are responsible for IMO. *Methanobrevibacter* smithii is the dominant microorganism in IMO. Interestingly, archaeon overgrowth can also occur in the colon and throughout the entire body. Archaeons use hydrogen produced in the gut that result from carbohydrate fermentation for methanogenesis. Excess Archaea, or methane-producing anaerobic organisms, occur in approximately 30% of small intestinal bacterial overgrowth patients [39,112]. Studies show a strong correlation between methane and irritable bowel syndrome with constipation. Exhaled methane levels are directly proportional to the degree of constipation [39,113,114]. In addition to constipation, IMO symptoms can include bloating, abdominal pain and decreased bowel motility. According to a study by Madigan et al. [112], IMO patients have different symptoms and clinical conditions than SIBO patients, and are less likely to exhibit vitamin B12 deficiency.

IMO diagnosis, as with SIBO, is based on an LBT or GBT, and a concentration of ≥10 ppm at any time during the test indicates methanogen colonisation.

Antibiotics are the most common treatment for IMO. In their research, Pimentel et al. [115] used a dose of 500 mg twice daily for ten days for patients with IBS and IMO. After repeat breath tests, it was found that 20% of patients in the study group exhibited reduced methane concentrations of ≥3 ppm compared to the control group (1%). A combination treatment with rifaximin and neomycin delivered better results. Low et al. [116] assigned patients to one of three study groups: treatment with neomycin alone at 500 mg twice daily for ten days, rifaximin for ten days at 400 mg three times daily, or a combination of rifaximin and neomycin for ten days. For 33% of those treated with neomycin alone, methane levels were reduced to undetectable levels (≥3 ppm). This was also true for 28% of those treated with rifaximin alone and 87% treated with both antibiotics.

## 6. Conclusions

People with gastrointestinal disorders, as well as other systemic diseases, may suffer from intestinal dysbiosis. Conditions synonymous with chronic inflammation are not without their consequences for the microbiota, but dysbiosis itself also disrupts the body’s homeostasis, which can influence the development of serious diseases. Microbial overgrowth reduces patients’ quality of life and causes discomfort, abdominal pains, bloating, gas, diarrhoea, and constipation. To date, SIBO is by far the most extensively studied condition. It can lead to malabsorption, resulting in nutritional deficiencies, anaemia or hypoproteinaemia. In addition to SIBO, SIFO also appears to be a real threat. People with weakened immune systems, particularly those infected suffering from HIV, cancer, diabetes or those using certain medications, and healthy people (for unknown reasons) are susceptible to it. IMO has only been partially researched, and, unlike SIBO, the overgrowth is caused by prokaryotic organisms—archaeons. *Methanobrevibacter* smithii is dominant. LIBO is a new concept that has not been explored yet, but it may be a health risk. Dysbiosis in the large intestine has not been thoroughly evaluated, particularly in patients with unexplained gastrointestinal symptoms. More clinical research is needed to determine the extent of this problem. Despite the recent interest in the gut microbiome and its disorders, more research is required to determine the pathophysiology, effective treatment, and prevention methods of small and large intestinal microbiota overgrowth. Therefore, it seems necessary to develop metagenomics and its fields—metatranscriptomics and metaproteomics. Future research may enable specific changes in compositional diversity to be used as biomarkers of health or particular diseases. It is important to pay attention to the occurrence of dysbiosis in other diseases coexisting with SIBO.

## Figures and Tables

**Table 1:** Changes in the intestinal microbiota depending on the type of antibiotics taken [7,35]. ↑ means increase in the number of bacteria, ↓ decrease in the number of bacteria.

Type of Antibiotic	Gut Microbiota			
	*Actinobacteria*	Bacteroidetes	*Firmicutes*	*Proteobacteria*
Macrolide	*Actinobacteria*↓	*Bacteroides*↑	*Firmicutes*↓	*Proteobacteria*↑
Clarithromycin	*Actinobacteria*↓	*Bacteroides*↑	*Firmicutes*↓	*Proteobacteria*↑
Vancomycin			*Lactobacillus*↓ *Clostridium*↓	
Ciprofloxacin	*Bifidobacterium*↓	*Alistipes*↓ *Bacteroides*↑	*Faecalibacterium*↓ *Oscillospira*↓ *Ruminococcus*↓ *Dialister*↓	
Clindamycin	*Bifidobacteriaceae*↓ *Lactobacillus*↓			

**Table 2:** Suggested antibiotics for treatment of small intestinal bacterial overgrowth [39]. Reprinted with permission from Ref. [39]. Copyright 2020 Wolters Kluwer Health, Inc.

Antibiotic	Recommended Dose	Efficacy
Nonabsorbable antibiotic		
Rifaximin	550 mg t.i.d.	61–78%
Systemic antibiotic		
Amoxicillin-clavulanic acid	875 mg b.i.d.	50%
Ciprofloxacin	500 mg b.i.d.	43–100%
Doxycycline	100 mg q.d. to b.i.d.	^a^
Metronidazole	250 mg t.i.d.	43–87%
Neomycin	500 mg b.i.d.	33–55%
Norfloxacin	400 mg q.d.	30–100%
Tetracycline	250 mg q.i.d.	87.5%
Trimethoprim-sulfamethoxazole	160 mg/800 mg b.i.d.	95%

^a^ In the study, no testing was performed to reassess small intestinal bacterial overgrowth, although all participants had other objective measures of improvement; q.d. (*lat. quaque die*)—once a day; b.i.d. (*lat. bis in die*)—twice a day; t.i.d. (*lat. ter in die*)—three times a day; q.i.d. (*lat. quater in die*)—four times a day.

## Data Availability

Not applicable.
